# A Challenging Case of Endoscopic Retrograde Cholangiopancreatography Due to an Intradiverticular Papilla

**DOI:** 10.7759/cureus.86383

**Published:** 2025-06-19

**Authors:** Mustafa Süveran, Can Boynukara, Recep Cecen, Gürhan Sisman

**Affiliations:** 1 Gastroenterology and Hepatology, Acibadem Altunizade Hospital, Istanbul, TUR; 2 Internal Medicine, Acibadem University, Istanbul, TUR; 3 Internal Medicine, Istanbul University-Cerrahpasa, Istanbul, TUR

**Keywords:** biliary tract endoscopy, choledochoduodenostomy, endoscopic retrograde cholangio-pancreatography, hot-axios, juxtapapillar diverticula

## Abstract

Periampullary diverticulum (PAD) is often encountered and associated with increased technical complexity and risk of procedural failure, particularly when the papilla is located within the diverticular lumen. We report a case of a 52-year-old male patient with biliary leakage secondary to metastatic colorectal carcinoma, in whom standard ERCP attempts failed due to the papilla’s position within PAD. Given the challenging anatomy, endoscopic ultrasonography (EUS) was employed to evaluate biliary anatomy and facilitate guided access. Utilizing a cautery-enhanced lumen-apposing metal stent (LAMS), a successful EUS-guided choledochoduodenostomy was performed under fluoroscopic guidance, resulting in adequate biliary drainage without complications. A follow-up at two weeks demonstrated clinical improvement (normalizing bilirubin levels) and resolution of the biliary leakage. This case illustrates the potential utility of EUS-guided lumen-apposing stent placement, specifically the Hot-Axios, as a minimally invasive alternative in difficult biliary interventions involving PAD, with implications for reducing procedural time and improving success rates in anatomically complex scenarios.

## Introduction

Juxtapapillary diverticulum, also known as periampullary diverticulum (PAD), refers to a diverticulum situated proximal to the major duodenal papilla at the site of the ampulla of Vater [1]. It is characterized by an outpouching of the duodenal wall in close proximity to the ampulla of Vater, where the common bile duct and pancreatic duct drain into the duodenum. It can exhibit variable sizes and clinical presentations, ranging from asymptomatic to symptomatic manifestations such as biliary colic, pancreatitis, or cholangitis. In rare cases, PAD can result in Lemmel’s syndrome, characterized by obstructive jaundice in the absence of stones or tumors [2]. During endoscopic retrograde cholangiopancreatography (ERCP), a juxtapapillary diverticulum poses challenges due to the altered anatomical configuration and associated technical complexities. The proximity of the diverticulum to the ampulla of Vater can impact the visualization and cannulation of the bile duct and pancreatic duct, potentially leading to procedural difficulties and necessitating specialized techniques for successful intervention. A juxtapapillary diverticulum is a challenging topic, especially for endoscopic retrograde cholangiopancreatography (ERCP). Due to anatomical considerations, the success rate of ERCP is diminished [3]. When ERCP cannot be performed, the clinician can use endoscopic ultrasonography (EUS)-guided interventions to provide relief to the biliary system. Herein, we present a case of challenging ERCP, which contributes to the growing literature on EUS-guided approaches in PAD-associated ERCP failure.

## Case presentation

**Figure 1:**
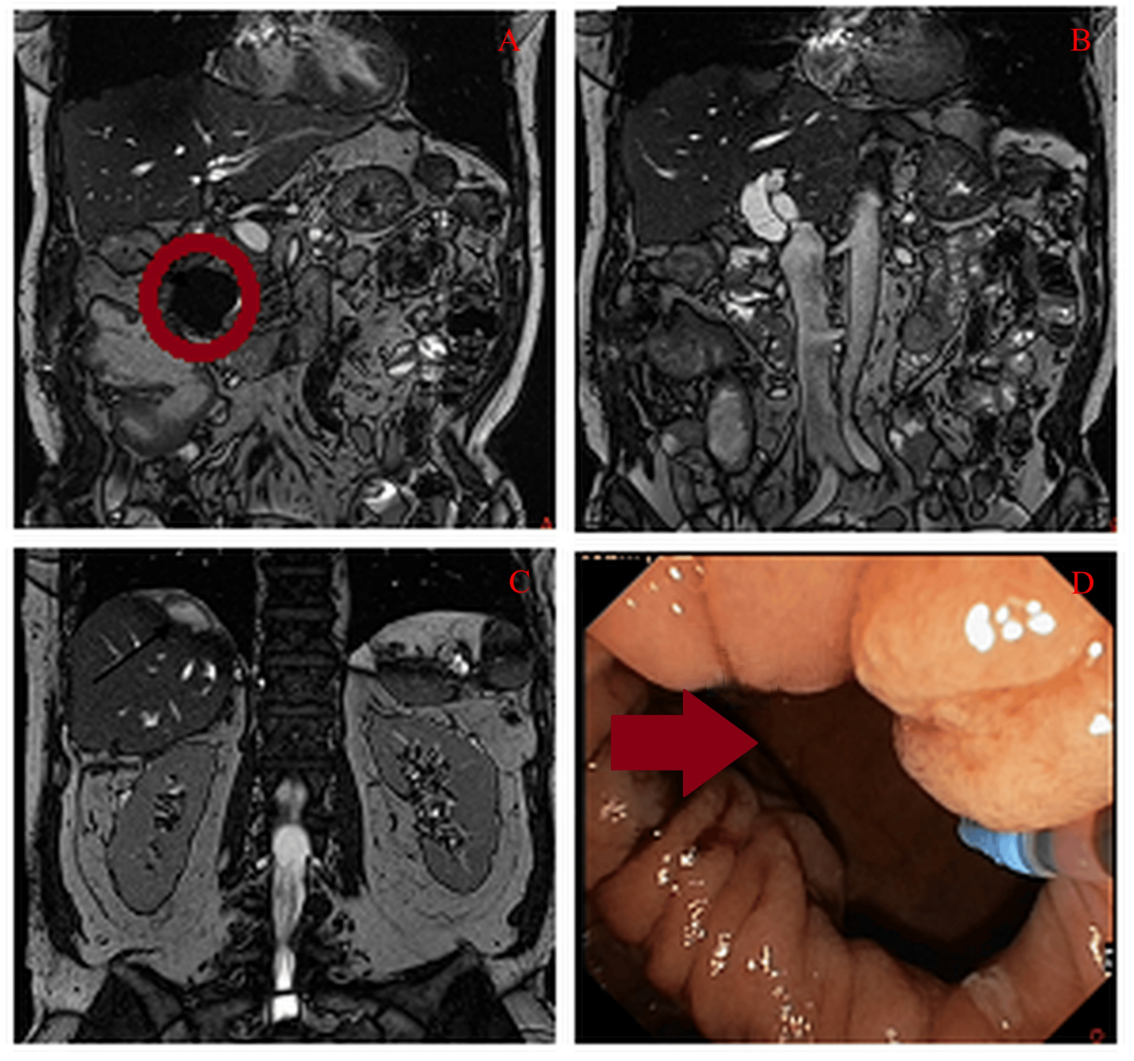
A: Duodenal diverticula (circle) in the MRCP image, B: Enlarged common bile duct (16mm) in the MRCP image, C: Bilioma and abscess in the MRCP image, D: Duodenal diverticula (arrow) and duodenal lumen (at the left bottom) MRCP: Magnetic resonance cholangiopancreatography

A 52-year-old male patient was referred to our clinic for evaluation of persistent biliary leakage and recurrent right upper quadrant pain. His medical history included metastatic colorectal carcinoma, initially managed with colon resection and adjuvant FOLFOX chemotherapy (5-fluorouracil, leucovorin, oxaliplatin). He had no chronic comorbidities or drug allergies. Surveillance imaging one year post-diagnosis revealed multiple liver metastases, prompting hepatic metastasectomy and segmentectomy (segments VI/VII) three months prior to presentation. Postoperatively, he developed complications, including biliary leakage and a subhepatic abscess localized to segment VII (Figure 1), confirmed on contrast-enhanced CT. Initial management involved percutaneous abscess drainage and intravenous piperacillin-tazobactam. The abscess culture revealed *Pseudomonas aeruginosa* infection. The abscess was drained and its size reduced at first, but then it re-enlarged. Laboratory investigations at this time demonstrated direct hyperbilirubinemia (total bilirubin: 8.8 mg/dL; RI: 1.3 mg/dL, direct: 6 mg/dL; RI: <0.3 mg/dL) and elevated acute-phase reactants (C-reactive protein (CRP): 280 mg/L, RI: <5 mg/dL), indicative of ongoing biliary obstruction and systemic inflammation (Table 1). Despite four weeks of conservative therapy, follow-up magnetic resonance cholangiopancreatography (MRCP) revealed persistent biliary leakage and enlargement of the abscess cavity (diameter: 6.5 cm vs. 4.2 cm initially).

**Table 1:** Reference intervals of the laboratory tests

Laboratory Test	Laboratory Values (Before Intervention)	Laboratory Values (2 weeks Later)	Reference Interval
Leukocyte Number	11.4 x10^3^/uL	6 x 10^3^/uL	4.06-10.6 x10^3^/uL
Haemoglobin	14.0 g/dL	13.8 g/dL	13.0-18.0 g/dL
Mean Corpuscular Volume	84.6 fL	85 fL	80.0-100.0 fL
Thrombocyte Number	186 x10^3^/uL	197 x10^3^/uL	150-439 10^3^/uL
Serum Creatinine	1.06 mg/dL	1 mg/L	0.7-1.3 mg/dL
Blood Urea Nitrogen	15 mg/dL	17 mg/L	6-20 mg/dL
C-Reactive Protein	280 mg/L	5 mg/L	<5 mg/L
Total Bilirubin	8.8 mg/L	1 mg/L	<1.3 mg/L
Direct Bilirubin	6 mg/L	0.3 mg/L	<0.3 mg/L

**Figure 2:**
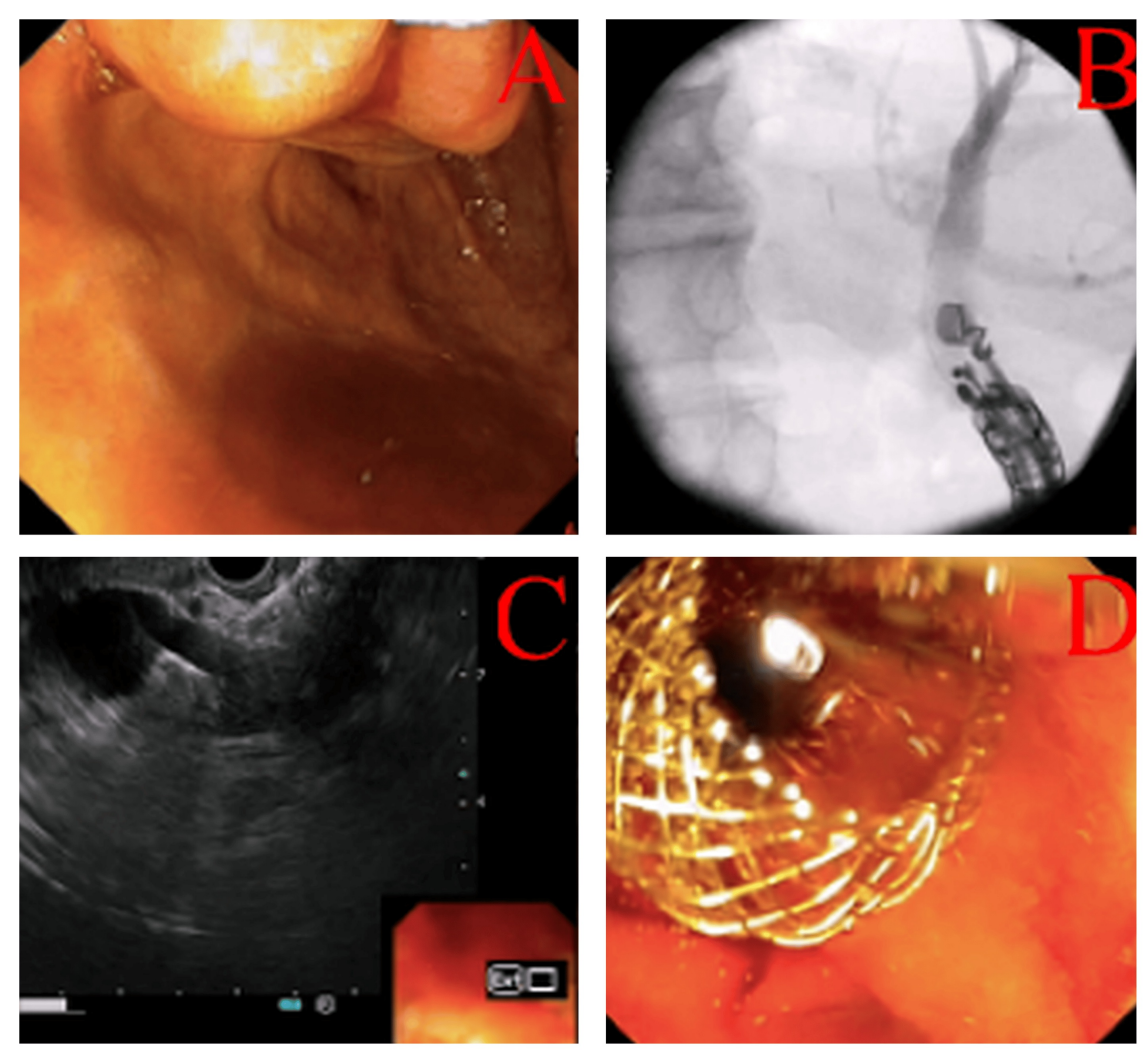
A: Cannulation trial of intradiverticular papilla at 1 o’clock, B: EUS-guided common bile duct cannulation under fluoroscopy, C: Hot-AxiosTM insertion of the common bile duct and acoustic artefacts, and D: Bile drainage inside of the choledochoduodenostomy line EUS: Endoscopic ultrasonography

The duodenoscopic evaluation identified a periampullary duodenal diverticulum with an intradiverticular papilla oriented at the 1 o’clock position (Figure 1), complicating ampullary visualization. Repeated attempts at conventional ERCP cannulation failed due to unstable scope positioning and inability to access the papilla within the diverticular cavity. Following procedural abandonment, anatomical reassessment via EUS demonstrated a markedly dilated common bile duct (CBD: 18 mm diameter) without evidence of choledocholithiasis or tumor recurrence. Under EUS and fluoroscopic guidance, a 6 mm × 8 mm cautery-enhanced Hot-Axios™ stent (Boston Scientific, Marlborough, MA, USA) was deployed, establishing a choledochoduodenostomy line (Figure 2). Immediate biliary drainage (BD) was confirmed fluoroscopically, with no procedural complications.

The patient reported rapid symptom resolution within 48 hours. At the two-week follow-up, laboratory markers showed normalization of bilirubin (total: 1.0 mg/dL, direct: 0.3 mg/dL) and a significant reduction in acute-phase reactants (CRP: 5 mg/L) (Table 1). Follow-up abdominal ultrasound confirmed resolution of the subhepatic abscess and stable stent positioning, with no evidence of residual biliary leakage.

## Discussion

This case underscores the evolving role of EUS-guided interventions in managing complex biliary complications, particularly in patients with altered anatomy and PAD. The patient, a 52-year-old man with metastatic colorectal cancer and a history of hepatic metastasectomy, developed persistent biliary leakage and abscess formation postoperatively. Despite optimal medical management, MRCP revealed progressive biliary leakage and abscess enlargement, necessitating definitive endoscopic intervention. Initial duodenoscopy identified a periampullary diverticulum with an intradiverticular papilla positioned at 1 o’clock, a configuration notorious for impeding standard ERCP cannulation due to obscured ampullary landmarks and unstable scope positioning. Repeated attempts at conventional biliary access failed, reflecting the challenges reported in the literature, where intradiverticular papilla and non-standard papillary orientations correlate with ERCP failure rates exceeding 30% [3-5]. 

The decision to transition to EUS-guided choledochoduodenostomy (CDS) was driven by the need for an alternative drainage route that bypassed the inaccessible papilla. EUS provided critical advantages in this scenario: it confirmed marked CBD dilation (18 mm), excluded tumor recurrence or lithiasis as contributors to the leakage, and enabled real-time visualization for precise transmural puncture. The deployment of a 6 mm x 8 mm cautery-enhanced Hot-Axios™ stent facilitated the procedure, combining electrocautery-mediated fistula creation and stent placement into a single step. This approach bypassed the anatomic challenges caused by the diverticulum and minimized procedural time and fluoroscopy exposure compared to traditional multi-stage techniques. The immediate establishment of choledochoduodenostomy resulted in effective biliary decompression, with clinical improvement evident at the two-week follow-up. 

The success of this intervention highlights several key considerations. First, EUS-guided drainage has emerged as a first-line rescue strategy after failed ERCP, particularly in patients with surgically altered anatomy or PAD, where conventional access is fraught with challenges. Second, using lumen-apposing metal stents (LAMSs) like Hot-Axios™ offers distinct advantages in such cases: their wide diameter ensures durable patency. At the same time, integrated electrocautery simplifies transmural access, reducing the risk of guidewire dislodgement or incomplete fistula formation. Notably, this patient’s CBD dilation (>15 mm) provided an optimal target for safe transmural intervention, aligning with guidelines that emphasize ductal diameter as a critical factor in EUS-BD success [6]. 

Importantly, this case also reflects broader trends in managing post-surgical biliary complications. Patients with hepatic resections, especially those involving segment VII, as in this case, are prone to biliary leaks due to disrupted ductal architecture and impaired healing [7]. While percutaneous drainage [8] and reoperation are historical alternatives, EUS-BD offers a minimally invasive, single-session solution with lower morbidity, particularly in frail or oncology patients. Furthermore, the avoidance of percutaneous catheters reduces the risk of tumor seeding in metastatic disease, a relevant concern in this patient’s context.

## Conclusions

This case demonstrates that EUS-guided BD using a cautery-enhanced Hot-Axios LAMS can be a safe and effective alternative for managing difficult ERCP cases complicated by periampullary diverticula with intradiverticular papillae. The technique offers the potential to reduce procedure time, improve success rates, and minimize complications in anatomically challenging scenarios where conventional approaches fail. In conclusion, this case reinforces the paradigm shift toward EUS-guided interventions as both rescue and primary strategies in complex pancreatobiliary disorders. The integration of advanced devices like Hot-Axios™ into therapeutic algorithms may redefine standards of care for periampullary diverticula and post-surgical biliary leaks, particularly when anatomic barriers preclude conventional approaches. Future studies comparing LAMS with alternative stents or techniques in similar cohorts will further refine patient selection and optimize outcomes in this challenging population. Further studies are warranted to establish the broader applicability and long-term outcomes of this innovative method.
